# Effects of 10 add‐on HF‐rTMS treatment sessions on alcohol use and craving among detoxified inpatients with alcohol use disorder: a randomized sham‐controlled clinical trial

**DOI:** 10.1111/add.16025

**Published:** 2022-09-07

**Authors:** Monja Hoven, Renée S. Schluter, Arnt F. Schellekens, Ruth J. van Holst, Anna E. Goudriaan

**Affiliations:** ^1^ Amsterdam UMC, Department of Psychiatry University of Amsterdam Amsterdam the Netherlands; ^2^ Amsterdam Institute for Addiction Research Amsterdam the Netherlands; ^3^ Donders Institute for Brain, Cognition, and Behavior, Department of Psychiatry Radboud University Medical Centre Nijmegen the Netherlands; ^4^ Nijmegen Institute for Scientist‐Practitioners in Addiction (NISPA) Nijmegen the Netherlands; ^5^ Center for Urban Mental Health University of Amsterdam the Netherlands; ^6^ Arkin and Jellinek Mental Health Care Amsterdam the Netherlands; ^7^ Amsterdam Public Health Research Institute Amsterdam the Netherlands

**Keywords:** Abstinence, alcohol use, alcohol use disorder, craving, neuromodulation, relapse, transcranial magnetic stimulation

## Abstract

**Background and aims:**

Alcohol use disorder (AUD) is a chronic disorder with high relapse rates. There are currently few clinical trials of high frequency repetitive transcranial magnetic stimulation (HF‐rTMS) to reduce alcohol use among AUD patients, and results are mixed. The current study tested the effect of 10 add‐on sessions of HF‐rTMS over the right dorsolateral pre‐frontal cortex (DLPFC) on alcohol use and craving.

**Design:**

Single‐center, single blind sham‐controlled parallel‐group RCT (*n* = 80), with 3 and 6 months follow‐up.

**Setting:**

Clinical treatment center in Amsterdam, the Netherlands.

**Participants:**

Eighty detoxified and abstinent AUD inpatients in clinical treatment (20 females, average age = 44.35 years).

**Intervention:**

Ten sessions of active or sham HF‐rTMS (60 10 Hz trains of 5 sec at 110% motor threshold) over the right DLPFC on 10 consecutive work‐days.

**Measurements:**

The primary outcome measure is the number of abstinent days over 6‐month follow‐up (FU). Secondary outcome measures are craving over 6‐month FU (alcohol urge questionnaire and obsessive‐compulsive drinking scale), time to first relapse over 6‐month FU and grams of alcohol consumed over 6‐month FU. Additional outcome measures: full abstinence over 6‐month FU and treatment success over 12‐month FU.

**Findings:**

HF‐rTMS did not affect the number of abstinent days over 6 months FU [sham = 124 ± 65.9 days, active = 115 ± 69.8 days, difference: 9 days, 95% confidence interval (CI) = Poisson model: 0.578–3.547]. Moreover, HF‐rTMS did not affect craving (AUQ/OCDS) (sham = 15.38/5.28, active = 17.48/4.75, differences = 2.1/−0.53, 95% CI mixed‐effects model = −9.14 to 2.07/−1.44 to 2.40).

**Conclusions:**

There was no clear evidence that high‐frequency repetitive transcranial magnetic stimulation over the right dorsolateral pre‐frontal cortex treatment has a long‐term positive effect on alcohol use or craving as add‐on treatment for alcohol use disorder. High treatment response at 6‐month follow‐up could have limited the possibility to find an effect.

## INTRODUCTION

Alcohol use disorder (AUD) is frequently a chronic relapsing disorder in those in addiction treatment, characterized by loss of control over intake, despite awareness of the harmful consequences [1]. Furthermore, a negative emotional state can arise when alcohol is not available [2, 3], and the mental health of the patient can be severely affected with comorbidities such as depression, anxiety and insomnia [4]. Often AUD patients experience intense urges or an irresistible desire to consume alcohol, also known as craving [5, 6, 7]. It is a world‐wide health problem, with 2.6% of the world population meeting criteria for AUD [8]. Each year in the Netherlands alone, already more than 30 000 individuals are hospitalized for the treatment of AUD [9].

Current AUD treatment mainly consists of psychological and pharmacological interventions [10]. Pharmacological treatment consists of anti‐craving medication, such as naltrexone and acamprosate [11, 12]. Some of the available psychological treatments are cognitive behavioral therapy (CBT) (focusing upon improving problem‐solving skills, enhancing drinking refusal skills and developing effective coping strategies by cognitive–behavioral treatment methods), motivational enhancement therapy (MET) (with a focus on enhancement of motivation to change behavior through motivational interviewing) and acceptance and commitment therapy (ACT) (which focuses upon acceptance of emotions and commitment to values that are set at the beginning of treatment) or contingency management (consisting of providing rewards when patients remain abstinent) [13]. The effectiveness of MET, CBT and ACT in alcohol and substance use disorder is small to moderate when compared to no treatment [14, 15, 16]. The effectiveness of contingency management is moderate when provided during treatment; however, this effect is abandoned after longer follow‐up periods [17, 18]. Despite these various treatment options, relapse rates are still approximately 60% 1 year after treatment [19, 20]. These high relapse rates indicate a need of novel treatment options for AUD.

From a neurobiological perspective, AUD is characterized by neuroplastic changes in several brain circuits. The executive function, reward and stress circuits are usurped by drugs of abuse, including alcohol, via multiple neurotransmitter‐specific neuroplasticity circuits [21]. For example, cue‐exposure can elicit recruitment of the reward circuit, which has been associated with subjectively experienced craving (reflected by increased neuronal activity in the reward circuit) [7]. Moreover, reductions in executive functioning in combination with decreased pre‐frontal cortex activity is often found in patients with AUD [21, 22]. This can lead to loss of control over the reward circuit, and thereby over impulses and behavior. Together, loss of control over the reward circuit and diminished executive control often results in issues managing craving and consequently relapse [1, 23].

Transcranial magnetic stimulation (TMS) has gained attention as a new treatment option for substance use disorders, including AUD [24], because it has the potential to interfere with the disturbed neurobiology of these conditions. With TMS, magnetic pulses are derived from an electromagnetic coil wherein an alternating current is running. When placed over the skull the magnetic pulse induces a current in the underlying neuronal tissue [25]. When pulses are applied in multiple trains, it is referred to as repetitive TMS (rTMS). Depending upon whether the frequency is low (LF) or high (HF), stimulation is inhibitory or excitatory, respectively [26, 27, 28].

In substance use disorder studies, HF‐rTMS is often applied over the dorsolateral pre‐frontal cortex (DLPFC) [29], as it is part of the executive function circuit [30]. The hypothetical effect of stimulation of these superficial brain regions is, first, to increase pre‐frontal cortex activity and strengthen the hypofunctional pre‐frontal cortex, thereby increasing executive control [31, 32]. Secondly, by indirect divergence into deeper structures of the brain, such as the nucleus accumbens (NAcc) and ventral tegmental area (VTA) due to direct connectivity between the DLPFC and the mid‐brain, rTMS influences subcortical neuronal activity [33]. The changes in activity in these areas may potentially decrease craving and decrease the chances for relapse [24, 32, 34]. rTMS has been shown to increase emotion regulation in AUD [35] and to influence functional connectivity in AUD [36]. Thus, neuromodulation of the DLPFC could primarily strengthen the functioning of the PFC by increasing executive control. Secondarily, it could modulate activity and dopamine release in subcortical areas such as the NAcc and VTA which could decrease craving. Together, this may ultimately lead to decreased relapse rates, when applied in clinical trials in AUD [1, 31].

Thus far, studies addressing the effect of HF‐rTMS treatment on clinical substance use outcome measures, including relapse and alcohol use, in AUD are non‐existent. This is surprising, as relapse is suggested as the primary outcome measure for clinical trials [29, 37]. However, previous studies on the effect of HF‐rTMS on craving in various substances are abundant (for reviews see [38, 39, 40]). Few studies focus upon alcohol as a substance of abuse. Some of these studies applied one single session of HF‐rTMS over the right DLPFC, which did not result in decreased craving [41, 42, 43]. Several studies applied multiple sessions of HF‐rTMS over the DLPFC. In one study, 10 sessions of HF‐rTMS over the right DLPFC resulted in decreased craving immediately after stimulation, but not at 4‐week follow‐up [44]. In addition, two other studies with 10 HF‐rTMS sessions (left DLPFC) [45] or 15 accelerated (multiple sessions on 1 day) HF‐rTMS sessions (right DLPFC) [43] did not observe reduced craving. Concluding, the effect of multiple sessions of HF‐rTMS over the DLPFC on craving is unclear, especially after a longer follow‐up period.

Considering the lack of studies addressing the effect on subsequent alcohol use, and the mixed results of HF‐rTMS on craving, the current study applied 10 add‐on sessions of HF‐rTMS over the right DLPFC. We included hospitalized abstinent AUD inpatients in a randomized sham‐controlled clinical trial design to test whether HF‐rTMS would lead to decreased alcohol use and less craving. We hypothesized that the HF‐rTMS add‐on treatment increases number of abstinent days and decreases experienced craving. Thus, we expected a higher number of abstinent days in the active group compared to the sham group. Furthermore, we expected lower craving levels in the active group compared to the sham group.

## METHODS

### Study design

In this single‐center, parallel, single‐blind RCT, the effects of 10 sessions of add‐on HF‐rTMS treatment on alcohol use and craving was assessed. We included 80 hospitalized abstinent recently detoxified AUD inpatients, which were randomly assigned (1:1) to either treatment as usual (TAU) plus 10 sessions of active HF‐rTMS treatment or TAU plus 10 sessions of sham HF‐rTMS treatment on 10 consecutive work‐days as add‐on treatment. TAU consisted of behavioral therapy, being either cognitive–behavioral therapy (CGT) or acceptance and commitment therapy (ACT). Some patients also received additional psychopharmacological treatment (see Table 1 for an overview). Three months (90 days), 6 months (180 days) and 12 months (360 days) after the final HF‐rTMS treatment sessions patients were contacted by telephone to assess outcome measures. As described in our protocol [46], the primary outcome measure was number of abstinent days over 6 months, secondary outcome measures included craving over 6 months, time until first relapse over 6 months and grams of alcohol consumed over 6 months. Additional outcome measures were complete abstinence over 6 months and treatment success over 12 months. This protocol was approved by the Medical Ethical Committee of the Academic Medical Centre Amsterdam (2015_064) and registered in the Netherlands Trial Register (NTR) (number 5291). The design of this study, and all the aimed primary and secondary outcome measures and analyses, have been described elsewhere in more detail [46]. For a schematic overview, see Supporting information, Figure S1.

**TABLE 1:** Sample characteristics.

	Active group (*n* = 40)	Sham group (*n* = 40)
Age (mean, SD)	44.95 (10.03)	43.75 (11.41)
Gender (male/female)	29/11	31/9
Handedness (right/left)	37/3	38/2
IQ (median, range)	83 (47–97)	84 (42–100)
Years of education (mean, SD)	7.50 (3.44)	7.24 (3.61)
MoCA score (> 27: 18–26)	26/13	30/10
Motor threshold (median, range)	50.00 (40–70)	54.50 (38–60)
Type of TAU (CGT/ACT)	37/3	36/4
Anti‐craving medication (yes/no)	15/25	12/28
Anti‐depressant medication (yes/no)	15/25	7/33
Sedative medication (yes/no)	3/37	1/39
Duration of problematic alcohol use in years (mean, range)	11 (2–36)	9 (1–36)
Number of DSM‐IV criteria fulfilled (median, range)	9 (3–11)	9 (4–11)
Number of previously followed AUD treatments (median, range)	3 (1–8)	2 (1–11)
PTSD (yes/no)	5/35	6/34
Cocaine dependence (yes/no)	9/31	5/35
Cannabis dependence (yes/no)	8/32	8/32

ACT, acceptance and commitment therapy; AUD, alcohol use disorder; CGT, cognitive behavioral therapy; DSM‐IV, Diagnostic and Statistical Manual of Mental Disorders, version 4; MoCA, Montreal cognitive assessment; PTSD, post‐traumatic stress disorder; SD, standard deviation; TAU, treatment as usual.

### Study sample

At time of recruitment all patients were abstinent and in treatment as inpatients at the Jellinek Addiction Treatment Centre in Amsterdam (the Netherlands). TAU consisted of 6 full‐time weeks of CBT or ACT, supplemented by emotion regulation training and motivational enhancement therapy during group sessions. Furthermore, patients received individual sessions with a psychologist, focusing upon comorbidities and other patients’ problems that occurred during treatment. Additional weekly CBT or ACT mentor sessions were given, focusing upon maintaining abstinence. Finally, pharmacotherapy was prescribed to a subset of patients (see Table 1). Recruitment took place from March 2016 to February 2019.

### Sample size

Sample size was pre‐registered [46]. Because at the time this study was the first, to our knowledge, to investigate the effect of multiple HF‐rTMS sessions on the number of abstinent days across 6 months, we could not perform a proper power analysis and our estimate was based upon previous rTMS studies which used craving as primary outcome that showed a moderate effect size. Hence, in this study, with the current sample size, a power of 0.8 and a moderate effect size, we should be able to detect a difference of 35 abstinent days between groups.

### Inclusion and exclusion

Inclusion criteria were as follows: (1) age between 20 and 65 years and (2) recent DSM‐IV diagnosis of alcohol dependence (i.e. less than 4 months after detoxification). Exclusion criteria were: (1) Montreal cognitive assessment (MoCA) [47] score below 10, (2) insufficient knowledge of the Dutch language, (3) current recreational drug use, (4) current DSM‐IV diagnosis of major depression, schizophrenia or another psychotic disorder and (5) HF‐rTMS contraindications (such as a history of epileptic seizures, metal implants near the head or use of the following medication: imipramine, amitriptyline, doxepine, nortriptyline, maprotiline, chlorpromazine, clozapine, foscarnet, ganciclovir, ritonavir, theophylline), based on Rossi *et al*. [28].

### Procedures

After the entire research procedure was explained to the patients, they all gave informed consent. Subsequently, screening for in‐ and exclusion criteria took place. When all inclusion and no exclusion criteria were met, the patient was enrolled into the study and subsequently randomized into either the active or sham HF‐rTMS treatment group. The randomization module was implemented using the data management system Castor EDC (Castor Electronic Data Capture, Ciwit BV, Amsterdam, the Netherlands, 2016), and was used to ensure concealed randomization. Randomization was conducted by the executive researcher without the patient present. Randomization was performed based on the stratification factors: anti‐craving medication (yes/no) and age (20–40/41–65 years). After allocation to one of the treatment groups, patients started the research procedure on 10 consecutive work‐days. During the first session (baseline), sample characteristics and craving questionnaires were assessed. Furthermore, stimulus location as well as intensity was determined, followed by the first HF‐rTMS treatment. During the second to ninth sessions, side effects of the treatment of the day before were assessed and subsequently HF‐rTMS treatment was applied. During the 10th session (post‐session), side effects of the treatment of the day before were assessed, followed by the HF‐rTMS treatment and assessment of the craving questionnaires. Three months (90 days), 6 months (180 days) and 12 months (360 days) after the final HF‐rTMS treatment sessions patients were contacted by telephone to assess alcohol use and craving. For a schematic overview of this procedure, see Supporting information, Figure S1.

### Intervention

The HF‐rTMS treatment was applied with a 70‐mm double air film coil (Magstim Co., Whitland, UK) and a Magstim Rapid2 stimulator (Magstim Co.). Stimulation parameters were as follows: 60 10‐Hz trains of 5 sec at 110% motor threshold (MT) over the right DLPFC [36]. Location of the right DLPFC (F4) was determined by means of the international 10–20 electroencephalogram (EEG) system [48]. Single‐pulse TMS over the motor cortex was used to determine the resting MT. When five of 10 stimuli resulted in a muscular (left abductor pollicis brevis) response of the thumb muscular abduction, this stimulation intensity was taken as MT. The actual treatment was given at an intensity of 110% of the MT. During the sham intervention identical procedures and stimulation parameters were applied; however, the coil was tilted 90° relative to the scalp.

### Measures

#### Pre‐intervention participant characteristics

The following sample characteristics were assessed: age, gender, handedness, intelligence quotient (IQ) (by means of the Dutch version of the adult reading test (NLV) [49], years of education, MoCA score [47], type of TAU (CGT/ACT), use of anti‐craving medication (naltrexone or acamprosate) at baseline (yes/no), use of anti‐depressant medication at baseline (yes/no), use of sedative medication at baseline (yes/no), duration of problematic alcohol use (years), number of DSM‐IV criteria fulfilled (11 in total), number of previously followed AUD treatments and the presence of comorbid disorders as measured by the mini‐international neuropsychiatric interview (MINI) [50].

#### Primary outcome

As described in Schluter *et al*. [46], the primary outcome measure was defined as the total number of abstinent days across the 6‐month follow‐up period (from days 1 to 180 after the final HF‐rTMS treatment). Patients indicated their number of abstinent days on the time‐line follow‐back (TLFB), which was assessed at 3‐ and 6‐month follow‐up. The number of abstinent days was calculated by summing the abstinent days, as indicated on the TLFB [51] at 3‐ and 6‐month follow‐up.

#### Secondary outcomes

As described in Schluter *et al*. [46], the secondary outcome measure ‘craving’ was measured by means of the alcohol urge questionnaire (AUQ) [52], assessed at baseline, post HF‐rTMS treatment and at 3‐ and 6‐month follow‐up. Higher AUQ scores signal higher craving levels. The additional measure regarding craving was measured with the short version of the obsessive–compulsive drinking scale (OCDS) [53], which contained five questions. Higher OCDS scores were indicative of higher craving levels.

The secondary outcome measures regarding alcohol use were as follows: (1) grams of alcohol consumed across the 6‐month follow‐up period (from days 1 to 180 after the final HF‐rTMS treatment). Patients indicated the day and quantity of the type of beverage they consumed on the TLFB during a telephone interview at 3‐ and 6‐month follow‐up; (2) number of days until first relapse, which was counted from the TLFB assessed at 3‐ and 6‐month follow‐up. Relapse was defined as a heavy drinking day (> 60 g alcohol per day for men/ > 40 g of alcohol per day for women) [54]. For details on censoring, see the Analyses subsection.

#### Additional outcomes

The additional measures of alcohol use were as follows: (1) full abstinence at 6‐month follow‐up, coded as a binary variable indicating whether or not a patient consumed any alcohol during the 6‐month follow‐up period. This measure was derived from the TLFB assessed at 3‐ and 6‐month follow‐up and (2) treatment success at 12‐month follow‐up. Patients were subdivided into one of two categories based on the last 30 days of the 12‐month follow‐up. The first category is successful treatment, including abstinence (no use of alcohol) and non‐excessive drinking [21 drinking days (14 for women) per 30 days, with a maximum of four glasses per day]. The second category is unsuccessful treatment, including excessive drinking (more than 21 drinking days and more than four glasses per day) [55].

#### Safety and tolerability

Patients were asked whether they experienced any discomfort or side effects after the previous stimulation session. Reported side effects were categorized based on a pre‐determined list of possible side effects (containing: headache, pain or beep in the ear, reduced hearing, fainting or epileptic seizure). Some patients reported a side effect that was not included in the pre‐determined list. These side effects were also registered (uncomfortable sensations at stimulation site after the stimulation and tiredness after stimulation).

#### Blinding

Blinding success was measured by asking patients whether they believed to have received active or sham treatment after the final HF‐rTMS treatment.

### Analyses

The R Environment (RStudio Team, 2015; RStudio, Inc., Boston, MA, USA), Statistical Package for Social Sciences (SPSS) version 25.0, 2017 (IBM Corporation, SPSS Statistics for Windows, version 25.0; Armonk, NY, USA) and JASP (JASP Team, 2019; University of Amsterdam, the Netherlands) were used for statistical analyses. *P*‐values < 0.05 were considered significant. Primary analyses followed the intent‐to‐treat principle.

#### Missing data

Due to the nature of our study design, we have various missing data patterns. Despite whether or not patients dropped out of treatment, all were called at 3‐, 6‐ and 12‐month follow‐up. Furthermore, when a participant did not answer the telephone during the 3‐month follow‐up, they were called at 6‐month follow‐up. Therefore, in some cases, data were not collected at 3‐month follow‐up, but at a later moment (6 months or later); for some patients we only have data at 3‐month follow‐up but not at 6‐month follow‐up. In those patients who were reached later, the craving questionnaires were not assessed as these were outdated, but measures derived from the TLFB were obtained. When patients were lost to follow‐up, but were later re‐admitted to Jellinek Addiction Treatment Center, they were then asked to fill in the TLFB. Again, in these cases no craving questionnaires were assessed.

**FIGURE 1:**
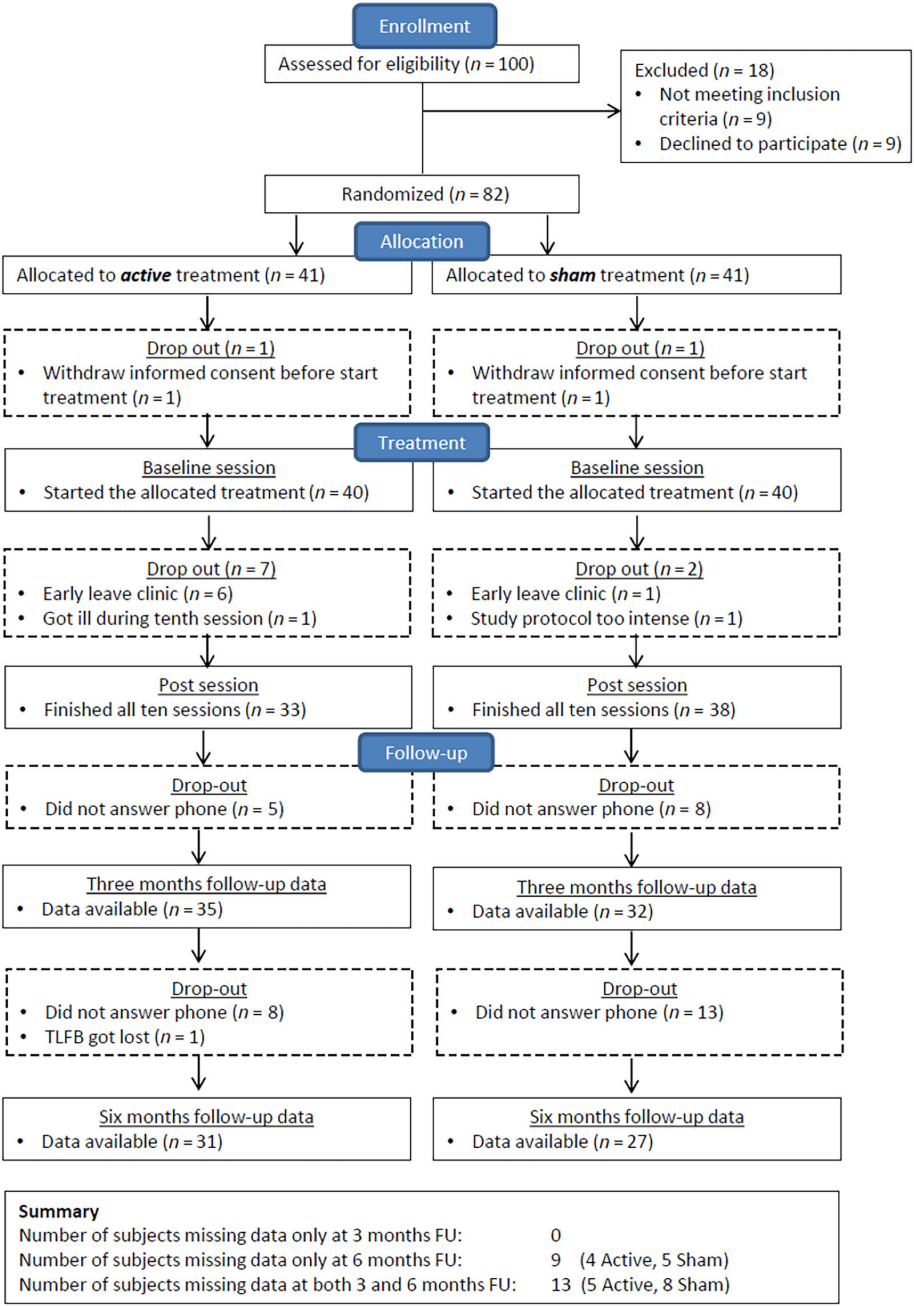
Consolidated Standards of Reporting Trials (CONSORT) flow‐chart. Overview of number of patients during enrollment, allocation and treatment

For an overview of missing data for the primary outcome measure, see Figure 1. For a flow diagram of follow‐ups for all secondary outcome measures see Supporting information, Figs S2 and S3. For an overview of missing data for the secondary and additional outcome measures, see Supporting information, Tables S1 and S2. As being lost to follow‐up could have various reasons (e.g. either changed telephone number to part from contacts related to their addictive disorder in the past, or relapsed to daily drinking), we assumed a missing at random (MAR) data process. For our primary intent‐to‐treat analyses we assumed a worst‐case scenario in which participants with missing data had returned to daily drinking, but have also performed additional complete case analyses in which we take into account the data as they are. In all analyses similar results were found, suggesting that our findings do not depend upon differences in missingness between groups.

#### Primary outcome

The primary outcome measure of total number of abstinent days across the whole 6‐month follow‐up period was calculated as a single value, where the abstinent days at 3‐ and 6‐month FU were added to gain a total number over 180 days. For our primary analysis, we assumed participants with missing data to have immediately relapsed after last contact, and thus entered zero abstinent days for their missing values at either 3‐ and 6‐month follow‐up, or both. To assess the effect of treatment (active versus sham) on the total number of abstinent days across the 6‐month follow‐up period, a generalized mixed‐effects model with a Poisson family and log link was implemented in the *afex* package [56]. The model contained the number of abstinent days as dependent variable and treatment group as fixed effect, with a random intercept for participant. *P*‐values were obtained using likelihood ratio tests within the *afex* pakage. Moreover, the *brms* package was used to perform Bayesian generalized multi‐level models in order to obtain the 95% credible interval for the treatment effect, using default settings for the prior [57]. The effect on number of abstinent days was tested with a Bayesian model from the Poisson family, with the number of abstinent days as dependent variable with treatment group as fixed effect and a random intercept per participant. As complete case analysis we performed a similar generalized mixed‐effects model, now using complete cases only, using similar methods. Moreover, to assess time × by group interactions, an additional mixed‐effects model was run, described in the Supporting information.

#### Secondary outcomes

First, to determine whether craving at baseline of the active group differed from the sham group, craving scores of the baseline session were compared between groups using a Mann–Whitney *U*‐test.

Linear mixed‐effects models were used to determine the effect of treatment (active/sham) and session (baseline/post/3‐month follow‐up/6‐month follow‐up) on craving. Two separate mixed‐effects models were built for the two dependent variables: AUQ and OCDS, for which we used all available data. For AUQ, the model included fixed effects of treatment and session, as well as the interaction term of treatment and session, and a random intercept of participant. Adding a random slope of session per participant improved model fit, which was assessed with χ^2^ tests on log‐likelihood, as well as by comparing the Akaike information criterion (AIC) values. For OCDS, the model included fixed effects of session and treatment and their interaction, as well as a random intercept of participant and random slope for session. For both models, *P*‐values were obtained using the Satterthwaithe approximation for degrees of freedom.

For secondary outcomes of alcohol use, the following analyses were performed. For the outcome measure of days until first relapse, we first censored the data. In case a participant did not relapse within the 6‐month follow‐up period, they were deemed censored cases with a time to event of 180. Participants who were lost to follow‐up were assumed to have experienced a relapse event immediately after last contact (either day 0 for participants with full missing data, or day 90 for participants with missing data at 6 months only). To assess the association between treatment group and days until first relapse, a Cox proportional hazards regression model was performed. Hazard ratios (HRs) with corresponding 95% CIs were calculated. Moreover, a complete‐case analysis was performed in which both participants who did not relapse within the follow‐up period were censored (at time‐to‐event of 180 days), as well as participants who were lost to follow‐up (and were censored either at time to event of 0 or 90 days). We performed an identical Cox proportional hazards regression model to compare treatment groups.

To determine the effects of treatment group and time on grams of alcohol we performed a linear mixed‐effects model with fixed effects of treatment group, time and their interaction. An additional complete case analysis was performed in which grams of alcohol consumed across 6 months was calculated by adding the values observed at 3‐ and 6‐month FU. Grams of alcohol were then compared between the groups using a Mann–Whitney *U*‐test due to non‐normality. Effect sizes for this test were obtained using the following formula: r=Z/√N.

#### Additional outcomes

For both treatment success and full abstinence we performed an intent‐to‐treat analysis in which we assumed all missing cases to have had (1) an unsuccessful treatment or (2) to not be abstinent. χ^2^ tests were performed to test for group differences (Fisher’s exact tests were used in case the expected counts were less than 5), and effect size was obtained by performing a Cramér’s V test. Additional complete case analyses were performed for both measures, for which we only used complete cases. Group differences were again tested using χ^2^ tests.

#### Safety and tolerability

To assess whether the reported side effects differed between the treatment groups, χ^2^ tests (or Fisher’s exact tests in case the expected counts were less than 5) were performed for each reported side effect separately. A *P*‐value of < 0.05 was considered significant.

#### Blinding

In order to determine whether blinding succeeded, percentage of individuals who guessed their treatment allocation correct was calculated. A binomial test was used to determine whether this percentage significantly differed from chance level (50%). A *P*‐value < 0.05 of was considered significant.

## RESULTS

### Sample characteristics

In total, 100 individuals were screened for eligibility, from whom 82 were included. Before the start of the add‐on treatment two individuals withdrew their informed consent. Therefore, 80 participants started the study (see Figure 1, Table 1).

### Dropout and data loss during treatment and follow‐up

As some participants were lost to follow‐up, we aimed to first compare the completeness of data regarding the primary outcome between the treatment groups, where we split up participants depending on whether they had complete or incomplete data on the primary outcome. A χ^2^ test showed that there were no differences in completeness of data between the treatment groups (nine incomplete cases in the active group, 13 incomplete cases in the sham group; χ^2^ = 0.5643, *P* = 0.452). Thus, importantly, there are no differences in dropout or data loss between the groups, which could potentially explain our further findings. Moreover, when testing for differences in baseline characteristics between the complete data and incomplete data group, no significant differences were found that survived multiple testing corrections.

### Primary outcome

#### Number of abstinent days

The primary intent‐to‐treat analysis using a generalized mixed‐effects model showed that treatment group did not predict the number of abstinent days (Table 2a). This means that the number of abstinent days across the 6‐month follow‐up period did not differ between the active treatment group [assuming participants with missing data to have immediately relapsed: mean = 115 days (~63.8%) ± 69.8 days] and the sham treatment group [assuming participants with missing data to have immediately relapsed: mean = 124 days (~68.8%) ± 65.9 days]. The complete case analysis showed similar results, as the number of abstinent days did not differ between the treatment groups [complete cases: active mean = 152 days (~84.4%) ± 41.3 days, complete cases: sham mean = 158 days (~87.8%) ± 30.6 days] (Table 2b).

**TABLE 2:** Results of the generalized linear mixed‐effects models regarding the effects of treatment group on the primary outcome measure of number of abstinent days.

(a) Analysis assuming participants with missing data to have returned to daily drinking							
Parameter	Estimate	SE	95% CI	Exponentiated estimate^a^	Exp. 95% CI	*Z*	*P*
Intercept	3.8548	0.3219	3.184 to 4.485	47.220	24.149 to 88.705	11.976	<0.001
Treatment group (active versus sham)	0.3512	0.4509	−0.548 to 1.266	1.421	0.578–3.547	0.779	0.436

(b) Complete case analysis							
Parameter	Estimate	SE	95% CI	Exponentiated estimate^a^	Exp. 95% CI	*Z*	*P*
Intercept	5.03320	0.06845	4.896 to 5.170	153.424	133.718–175.890	73.531	<0.001
Treatment group (active versus sham)	−0.06665	0.0938	−0.255 to 0.120	0.936	0.775 to 1.128	−0.711	0.477

(a) Results of the analysis assuming that participants with missing data have returned to daily drinking. (b) Complete case analysis in which only participants with data at 3 and 6 months’ follow‐up were included.

^a^ As the models use a log‐link function, the exponentiated estimate refers to a ratio.

CI, confidence interval; SE, standard error.

The Bayesian analysis showed that the 95% credible interval of the differences between the posterior distributions of the treatment groups spanned zero [95% credible interval = (−0.13, 0.26)]. This is a strong indication that there are no group differences in the number of abstinent days.

### Secondary outcomes

#### Craving

**FIGURE 2:**
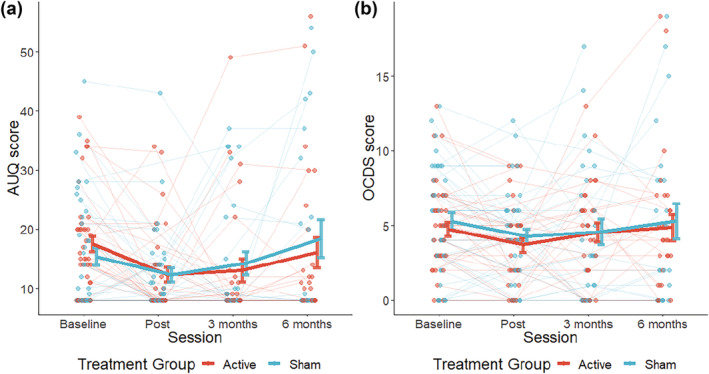
Graphs of the treatment effect for the (a) alcohol urge questionnaire (AUQ) and the (b) short version of the obsessive–compulsive drinking scale (OCDS). The baseline measures were assessed during the first test day, the post measure was assessed during the 19th test day and the 3 and 6 months were assessed during a follow‐up telephone interview. Points represent mean questionnaire scores per treatment group and session. Dots represent individuals and lines highlight within‐subject variation across sessions. Error bars represent group mean ± standard error of the mean (SEM)

The active treatment group (mean = 17.48 ± 8.376) and the sham treatment group (mean = 15.38 ± 9.513) did not significantly differ in mean AUQ score at baseline (*U* = 642.500, *P* = 0.122), as revealed by the Mann–Whitney *U*‐test. The linear mixed‐effects model did not show a significant effect of time, treatment group or a significant interaction between time and treatment group on AUQ score (Table 3, Figure 2a).

**TABLE 3:** Results of the linear mixed‐effects models regarding the effects of treatment group, session and their interaction on craving (AUQ and OCDS).

AUQ					
Parameter	Estimate	SE	95% CI	*t*	*P*
Intercept	16.458	1.9856	12.526 to 20.414	8.289	<0.001
Treatment group	−3.531	2.8252	−9.147 to 2.066	−1.250	0.215
Session	−0.674	0.9199	−2.522 to 1.143	−0.733	0.466
Session × treatment group	1.536	1.3195	−1.078 to 4.170	1.164	0.248

OCDS					
Parameter	Estimate	SE	95% CI	*t*	*P*
Intercept	4.239	0.678	2.898 to 5.592	6.253	<0.001
Treatment group	0.488	0.966	−1.436 to 2.402	0.505	0.615
Session	0.0934	0.298	−0.503 to 0.683	0.316	0.753
Session × treatment group	0.028	0.429	−0.821 to 0.888	0.066	0.948

AUQ, alcohol urge questionnaire; CI, confidence interval; OCDS, obsessive–compulsive drinking scale; SE, standard error.

A two‐sample *t*‐test did not reveal a significant difference between the active treatment group (4.75 ± 3.019) and the sham treatment group (5.28 ± 3.630) in OCDS score at baseline (*t*_(_ _77__)_ = − 0.703, *P* = 0.484). The linear mixed‐effects model did not show a significant effect of time, treatment or a significant interaction between session and treatment on OCDS score (Table 3, Figure 2b).

#### Grams of alcohol

The mixed‐effects model showed that there was no effect of group, a significant effect of time (showing more grams of alcohol over time), but no interaction effect between group and time on grams of alcohol consumed (Table 4a). Our second analysis, using a Mann–Whitney *U*‐test, also showed no significant differences in complete cases in terms of grams of alcohol consumed between the active treatment group and the sham treatment group, with an effect size of 0.0587, indicating that 5.8% of the variance in grams of alcohol consumed was explained by type of add‐on treatment (Table 4b).

**TABLE 4:** Results of the secondary and additional measures of alcohol use.

(a) Grams of alcohol					
Parameter	Estimate	SE	95% CI	*t*	*P*
Intercept	1420.53	838.79	−219.74 to 3060.80	1.694	0.095
Treatment group (active versus sham)	820.15	1160.53	−1449.28 to 3089.59	0.707	0.482
Time	1071.86	498.66	94.95 to 2048.89	2.149	0.036
Treatment group × time	−535.61	687.52	−1897.54 to 802.81	−0.779	0.439

(b) Grams of alcohol complete case analysis					
Parameter	Mann–Whitney *U*	*P*	Effect size + CI		
Treatment group	437.50	0.599	0.0587 [−0.176 to 0.271]		

(c) Days until first relapse					
Parameter	Hazard ratio	CI	*Z*	*P*	
Treatment group	0.9014	0.5476–1.484	−0.408	0.683	

(d) Days until first relapse complete case analysis					
Parameter	Hazard ratio	CI	*Z*	*P*	
Treatment group	1.0935	0.615–1.945	0.304	0.761	

(e) Full abstinence					
Parameter	χ^2^	d.f.	Effect size (V)	*P*	
Treatment group	0.078	1	0.031	0.7799	

(f) Full abstinence complete case analysis					
Parameter	χ^2^	d.f.	Effect size (V)	*P*	
Treatment group	0.088	1	0.036	0.767	

(g) Treatment success					
Parameter	Odds ratio	95% CI	Effect size (V)	*P*	
Treatment group	1.242	0.450–3.469	0.026	0.815	

(h) Treatment success complete case analysis					
Parameter	Odds ratio	95% CI	Effect size (V)	*P*	
Treatment group	1.520	0.213–12.366	0.014	0.691	

CI, confidence interval; d.f., degrees of freedom; SE, standard error.

#### Days until first relapse

**FIGURE 3:**
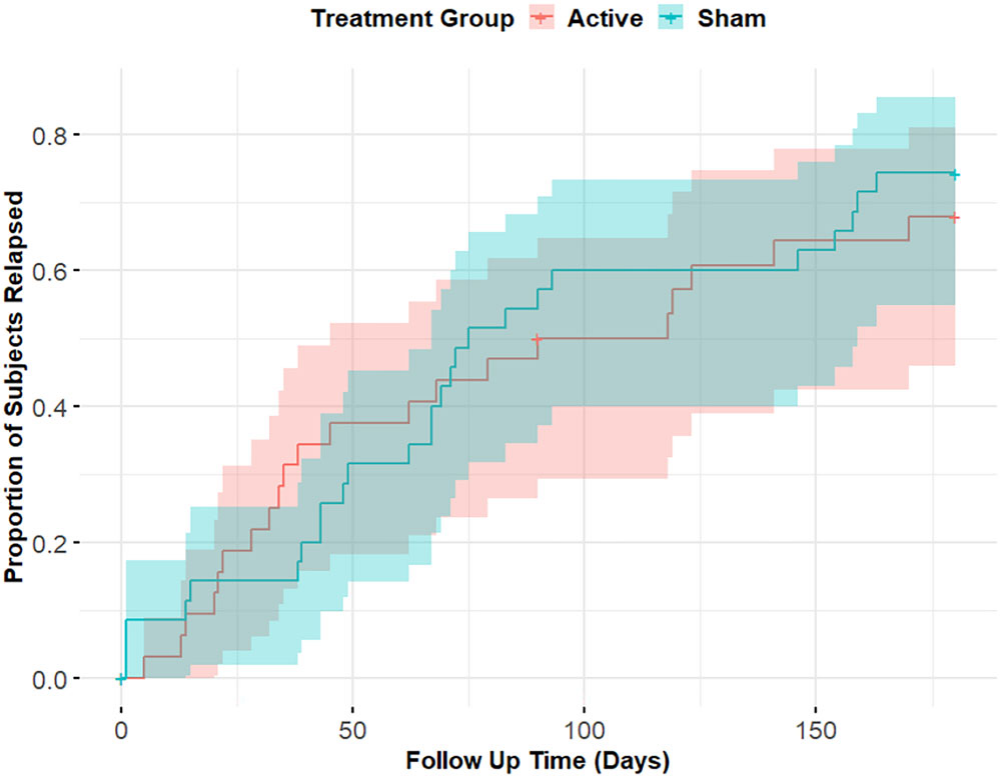
Kaplan–Meier plot of relapse [relapse was defined as a heavy drinking day (> 60 g alcohol per day for men/> 40 g of alcohol per day for women)] in both treatment groups. The *x*‐axis represents follow‐up time in days after the treatment and the *y*‐axis shows the proportion of participants who have experienced a relapse. The upward trend indicated that over time more participants (a larger proportion) experienced a relapse. The red line represents the active treatment group, whereas the blue line represents the sham treatment group. No differences were found between the treatment groups

The Cox regression showed that days until first relapse did not significantly differ between the active treatment group and the sham group, showing that there was no increased hazard for relapse in the sham group (Table 4c). The additional regression on complete cases showed similar results, with no differences between the treatment groups (Table 4d, Figure 3).

#### Additional measures

##### Full abstinence

The χ^2^ test in the intent‐to‐treat analysis did not show a significant difference in full abstinence between the active treatment group (nine reached full abstinence, whereas 31 did not) and the sham treatment group (seven reached full abstinence, whereas 33 did not) (Table 4e). Similar results were found with complete case analysis (Table 4f). The effect size indicated that approximately 3% of the variance in full abstinence rate was explained by an effect of add‐on HF‐rTMS (Table 4f).

##### Treatment success

Furthermore, Fisher’s exact test showed in the intent‐to‐treat analysis that treatment success (measured at 12‐month follow‐up) did not differ significantly between the active treatment group (and the sham treatment group (Table 4g). Similar results were found with complete case analysis (Table 4h). Effect size showed that approximately 2% of the variance in treatment success was explained by an effect of the add‐on HF‐rTMS treatment.

### Safety and tolerability

Patients in the active group reported headache after stimulation, pain or beep in the ear, tiredness after stimulation and uncomfortable sensation at the stimulation site after stimulation as side effects. Patients in the sham group reported the same side effects, except for pain or beep in the ear. The sham HF‐rTMS group reported more headache (χ^2^_(1)_ = 4.477, *P* = 0.034), while the active group reported more uncomfortable sensations at the stimulation site after stimulation (χ^2^_(1)_ = 4.407, *P* = 0.036) compared to the sham treatment group. Occurrence of the other side effects was not significantly different between the active stimulation group and the sham stimulation group (see Supporting information, Table S3).

### Blinding

In total, 68 patients reported their treatment beliefs, of which 39 patients (57,4%) believed to have received active treatment. Approximately two‐thirds of participants (63.2%) guessed their treatment allocation accurately. The binomial test revealed that the proportion of patients who guessed their treatment allocation correctly (0.63) was significantly (*P* = 0.038) higher than chance level (0.50).

## DISCUSSION

The aim of the current study was to test whether 10 add‐on sessions of HF‐rTMS over the right DLPFC of hospitalized abstinent AUD patients would reduce alcohol use and diminish experienced craving. Results of the current study did not show significant differences in alcohol use between the active and the sham group at 6‐month follow‐up, nor a significant difference in treatment success at 12‐month follow‐up. Furthermore, results of the current study did not show an effect of session or treatment group, nor an interaction of the two, on experienced craving levels. This suggests no beneficial add‐on effect of active HF‐rTMS treatment compared to sham treatment on relapse and craving in AUD patients receiving treatment as usual. This was confirmed by low effect sizes and Bayesian analyses indicating lack of effects and moderate evidence for the null hypotheses (i.e. no effect of HF‐rTMS treatment).

The current study was one of the first, to our knowledge, to address the effect of HF‐rTMS treatment on alcohol use outcome measures such as number of abstinent days and relapse, which is surprising, as relapse is suggested as primary outcome measure of clinical trials [29, 37]. Contrary to our hypotheses we did not find evidence for a beneficial effect of HF‐rTMS add‐on treatment on alcohol use and relapse rates.

First, regarding alcohol use, very few studies addressed the effect of HF‐rTMS on alcohol use and have found mixed results. A small study (*n* = 17) using HF‐rTMS on the left DLPFC showed no changes in post‐treatment or 1 month follow‐up number of drinks between treatment groups [58]. Another relatively small RCT (*n* = 30) using HF‐rTMS on the right DLPFC showed a group effect on post‐treatment alcohol use with lower alcohol use in the active group, but no group × time interaction analyses, were reported using follow‐up measures of alcohol use [59]. Other methods of applying rTMS might be more effective in improving alcohol use, such as deep rTMS using an H‐coil [60], used deep rTMS over the medial PFC in a small sample (*n* = 18) in a pilot study and found a decrease in the number of drinks in the active stimulation group (*n* = 9) from baseline to post‐treatment and up to 3 months (however, note that five patients dropped‐out at 3‐month follow‐up and only data of four patients were analyzed), and a significant reduction in number of drinks in the sham group (*n* = 9) from baseline to 1‐month follow‐up. However, no direct statistical test was performed to compare number of drinks between groups over time in terms of an interaction effect (a group × time interaction effect). Using separate *t*‐tests does not provide correct information about the group × time interaction effect of interest, and instead a repeated‐measures analysis approach should be used to enable valid conclusions to be drawn [61]. Another small pilot study (*n* = 11) using deep rTMS on the bilateral DLPFC found that drinking behavior improved in the active treatment group, but not in the sham group [62]. However, again no direct statistical tests were performed to test for a group × time interaction effect. Both these studies were pilot studies, and thus results are preliminary. A larger study targeting the insula using deep rTMS in 56 AUD patients showed no evidence of a group × time interaction on alcohol use, and thus showed no effect of the active over the sham condition on alcohol use over a period of 12 weeks’ follow‐up [63]. A recent proof‐of‐concept study (*n* = 51) showed that dTMS targeting mid‐line frontal areas reduced heavy drinking days in the active versus sham condition over a period of 12 weeks without an interaction effect with time [64]. Overall, most studies reported a lack of a positive effect of HF‐rTMS add‐on treatment on drinking behavior and alcohol use in the long term, which is in line with the findings of this larger RCT. Studies addressing the effect of HF‐rTMS over the DLPFC as add‐on treatment in other substances of abuse also report mixed results. For example, two studies indicated an acute reduction of cigarette consumption [65, 66] and one study reported higher abstinence from smoking at 3‐month follow‐up [67]. Furthermore, two pilot studies in cocaine use disordered patients showed mixed results, one indicating acute beneficial effects of HF‐rTMS treatment compared to TAU [68], but the other not reporting long‐term (6 months) effects of HF‐rTMS treatment compared to sham [69]. Together, our findings are in line with the negative findings reported in the relatively larger studies in AUD [63] and studies conducted in nicotine use disorder [65] and cocaine use disorder [69]. However, our findings are contrary to other smaller studies that were either pilot studies [60, 62] or did not report on follow‐up measures of alcohol use [59]. Furthermore, results of the current study are hard to compare to other studies as they did not include follow‐up periods [66, 68]. Hence, currently, there is limited evidence to suggest that HF‐rTMS treatment has a positive clinical treatment impact in AUD; however, in nicotine use disorder, the first results are more promising [67].

With regard to craving, the current study also did not find an effect of 10 HF‐rTMS sessions on acute and long‐term (up to 6 months) experienced craving. These results are in line with earlier studies showing no evidence for an effect of HF‐rTMS on craving in AUD patients. A relatively small RCT (*n* = 19) showed no acute craving differences between active and sham rTMS on the left DLPFC [70]. Moreover, multiple trials with sample sizes ranging from 24 to 31 participants showed no effects of rTMS on the right DLPFC on acute craving [41, 42, 43]. Del Felice and colleagues [58], in a small sample (*n* = 17) showed no improvements of acute craving or craving after 1‐month follow‐up using rTMS on the left DLPFC [58]. A larger study (*n* = 39 patients) using rTMS on the right DLPFC also failed to find an acute effect on craving [35]. Contrary to these null findings, two studies using rTMS on the right DLPFC reported positive results for craving: Mishra and colleagues reported reduced acute craving, whereas Belgers and colleagues showed a group × time interaction, showing decreased craving in the active group up to 3 months’ follow‐up [44, 59]. Differences in study samples could have had an influence on the outcomes, as Belgers *et al*. [59] collected data from outpatients whereas the current study only included inpatients. Other neuromodulation methods also show mixed findings in AUD; no effects of theta burst stimulation (TBS) targeting the medial PFC were found on acute craving (*n* = 24) [71] and, similarly, no effects on acute craving were found using TBS targeting the ventromedial PFC (*n* = 24) [72]. Also, a lack of effect on acute craving using deep TMS was found by Addolorato and colleagues [62]. Deep TMS over the insula also failed to improve craving over a 12‐week follow‐up period in a fairly large sample (*n* = 56) [63]. However, Ceccanti and colleagues showed a decrease in craving in the active condition up to 1‐month follow‐up which they did not find in the sham condition, but again did not directly test the group × time interaction effect [60, 61]. Finally, Harel and colleagues [64] found that mid‐line dTMS reduced acute craving without any long‐term follow‐up effects.

Several studies in other substances (cocaine and methamphetamine) have shown acute effects of HF‐rTMS on craving [73, 74]; however, these effects were not found at 6‐month follow‐up [68]. Therefore, future studies with longer‐term craving measures are required in order to be able to conclude whether or not HF‐rTMS add‐on treatment may have beneficial effects in reducing experienced craving.

Despite the absence of effect in our study, the potential effectiveness of rTMS in AUD could be studied with other parameters. Other methods of applying rTMS might be more promising for substance abuse, such as deep rTMS [60, 62, 63]. First, deep rTMS also resulted in decreased acute craving in patients with cocaine use disorder; however, no longer follow‐up periods were conducted [75]. Secondly, changing the target location may result in increased effectiveness of the HF‐rTMS treatment. The one study that reports long‐term beneficial effects on abstinence rates in nicotine dependence stimulated the left instead of the right DLPFC [67]. This is in line with a meta‐analysis that found favorable acute effects only for left‐sided DLPFC stimulation [76]. However, right‐sided stimulation has previously also been found to tend to have a bigger effect size [77], and another meta‐analysis found that active stimulation was only favorable over sham stimulation in trials focusing on the right DLPFC [78]. Thirdly, the method used to assess craving might also determine whether an effect is found. For example, studies that applied rTMS after provoking craving by showing pictures of substance‐related cues [74], touching instruments related to use or smelling and using fake substances [73] found acute beneficial effects on experienced craving. Finally, increasing the number of stimulation sessions might determine the impact of the rTMS treatment. In comparison to the treatment of depressive disorder (the standard treatment consists of 30 treatment sessions, followed by a maintenance period) [79], the current protocol (10 sessions) may have not included a sufficient number of stimulation sessions. Future studies must determine whether other stimulation techniques, other target areas, provoking craving before stimulation or increasing the number of stimulation sessions result in a positive effect of HF‐rTMS treatment on relapse or experienced craving. These studies should focus particularly on the long‐term effects.

Results of the current study might be affected by the selection of our outcome measures and timing of assessment. For example, absolute abstinence rates are low; however, the number of abstinent days is high. This suggests that relapse duration is short. Moreover, the TAU seems to have had a high treatment response, as complete cases show a high percentage of abstinent days which could have caused a ceiling effect. Furthermore, perceived craving was relatively low in our studied sample, which might be explained by the intake of anti‐craving medication in 27 of the 80 patients. These factors could potentially have affected our findings.

The results of the current study should be seen in the light of several limitations. For instance, in the current study, relapse as well as craving were measured by means of self‐report. This required patients to remember whether they have been drinking, what day and how much for the past 3 months, which might appear to be unreliable. However, in practice, patients were very well able to recall when, and how much, they were drinking [80, 81]. In addition, in the current sham condition, wherein the coil is tilted away from the scalp, stimulation of neuronal tissue does not take place, but also the somatosensory effect of stimulation is removed [82]. Indeed, unpleasant sensations of the skin after stimulation occurred less in the sham group. As a result, patients were able to identify above chance level to which of the treatment groups they were allocated. Although results of the current study were not affected by this, as no placebo response was detected, future studies should consider using a sham coil [83] which mimics somatosensory effects of stimulation. Moreover, the single ‐blind design could also be considered a limitation; however, minimal to no influence is expected on the outcome measure as the outcome measures were self‐reports on which the researcher had no influence.

Taken together, we found no evidence of an effect of 10 sessions of HF‐rTMS treatment over the right DLPFC on relapse rates and experienced craving in hospitalized AUD patients. These findings suggest that HF‐rTMS over the right DLPFC might not have added value in the treatment of these patients, despite some indications of efficacy in nicotine use disorder [67]. Future studies must determine whether other means of measuring craving, other stimulation techniques and other target areas do improve relapse rates and experienced craving.

## CLINICAL TRIAL REGISTRATION DETAILS

This clinical trial is registered in the Netherlands Trial Register, trial number 5291: https://www.trialregister.nl/trial/5151.

## DECLARATION OF INTERESTS

None.

## AUTHOR CONTRIBUTIONS

**Monja Hoven:** Data curation; formal analysis; investigation; project administration; visualization. **Renée Schluter:** Conceptualization; data curation; formal analysis; investigation; methodology; project administration; validation. **Ruth van Holst:** Conceptualization; data curation; formal analysis; investigation; methodology; supervision; validation. **Anneke Goudriaan:** Conceptualization; funding acquisition; methodology; supervision.

## Supporting information


**Figure S1:** Schematic overview of the study procedure.
**Figure S2:** Overview of drop‐out and data loss for each secondary outcome measure during follow‐up for the active rTMS treatment group. Note that the numbers of available data in this flow chart do not necessarily compare to our numbers of missing data in Table 1 and 2 in the main manuscript. For example, it is possible that a subject relapsed within the first three months follow‐up, which we assessed at the three months follow‐up time point, and then was lost to follow‐up at six months. For this outcome measure that would mean that we have complete information, but he/she would still be lost to follow‐up at six months.
**Figure S3:** Overview of drop‐out and data loss for each secondary outcome measure during follow‐up for the sham rTMS treatment group.
**Table S1:** Overview of missing data patterns for the secondary alcohol use outcome measures per follow up period and treatment group. Grams alcohol, days until first relapse and full abstinence were assessed at 3 and 6 months follow‐up, whereas treatment success was measured at 12 months follow up. Subjects that miss data of days until first relapse or full abstinence at 6 months did not have a relapse event and stayed abstinent for the first 3 months. NA: Not Applicable.
**Table S2:** Overview of missing data patterns for the secondary craving outcome measures per follow up period and treatment group. Craving was assessed pre‐treatment, post‐treatment, at 3 and 6 months follow up, resulting in various missing data patterns.
**Table S3:** Side effects reported in the rTMS and sham treatment groups.
