# Supplementary material for: Effects of 10 add‐on HF‐rTMS treatment sessions on alcohol use and craving among detoxified inpatients with alcohol use disorder: a randomized sham‐controlled clinical trial
Source: Addiction. 2022 Sep 7;118(1):71–85. doi: 10.1111/add.16025 (PMC10087396; doi:10.1111/add.16025)
Supplement: Supplementary file 1 — Figure S1: Schematic overview of the study procedure. Figure S2: Overview of drop‐out and data loss for each secondary outcome measure during follow‐up for the active rTMS treatment group. Note that the numbers of available data in this flow chart do not necessarily compare to our numbers of missing data in Table 1 and 2 in the main manuscript. For example, it is possible that a subject relapsed within the first three months follow‐up, which we assessed at the three months follow‐up time point, and then was lost to follow‐up at six months. For this outcome measure that would mean that we have complete information, but he/she would still be lost to follow‐up at six months. Figure S3: Overview of drop‐out and data loss for each secondary outcome measure during follow‐up for the sham rTMS treatment group. Table S1: Overview of missing data patterns for the secondary alcohol use outcome measures per follow up period and treatment group. Grams alcohol, days until first relapse and full abstinence were assessed at 3 and 6 months follow‐up, whereas treatment success was measured at 12 months follow up. Subjects that miss data of days until first relapse or full abstinence at 6 months did not have a relapse event and stayed abstinent for the first 3 months. NA: Not Applicable. Table S2: Overview of missing data patterns for the secondary craving outcome measures per follow up period and treatment group. Craving was assessed pre‐treatment, post‐treatment, at 3 and 6 months follow up, resulting in various missing data patterns. Table S3: Side effects reported in the rTMS and sham treatment groups. [file ADD-118-71-s001.docx]

**Supplementary Informatio**

**Effects of ten add-on HF-rTMS treatment sessions on alcohol use and craving among detoxified inpatients with alcohol use disorder: a randomized sham-controlled clinical trial**

List of authors: M. Hoven^1^*, R. S. Schluter^1^*, A. F. Schellekens^3^, R. J. van Holst^1,2^, A. E. Goudriaan^1,2,4^

*Contributed equally: shared first authorship

Authors affiliations and addresses:

1: Amsterdam Institute for Addiction Research, Amsterdam UMC, Department of Psychiatry, University of Amsterdam, Meibergdreef 5, 1105 AZ, Amsterdam, The Netherlands.

2: Center for Urban Mental Health, University of Amsterdam

3: Radboud University Medical Centre, Donders Institute for Brain, Cognition, and Behavior, Department of Psychiatry, Postbus 9101, 6500 HB, Nijmegen, The Netherlands.

4: Arkin and Jellinek, Mental Health Care, Klaprozenweg 111, 1033 NN, Amsterdam, The Netherlands.


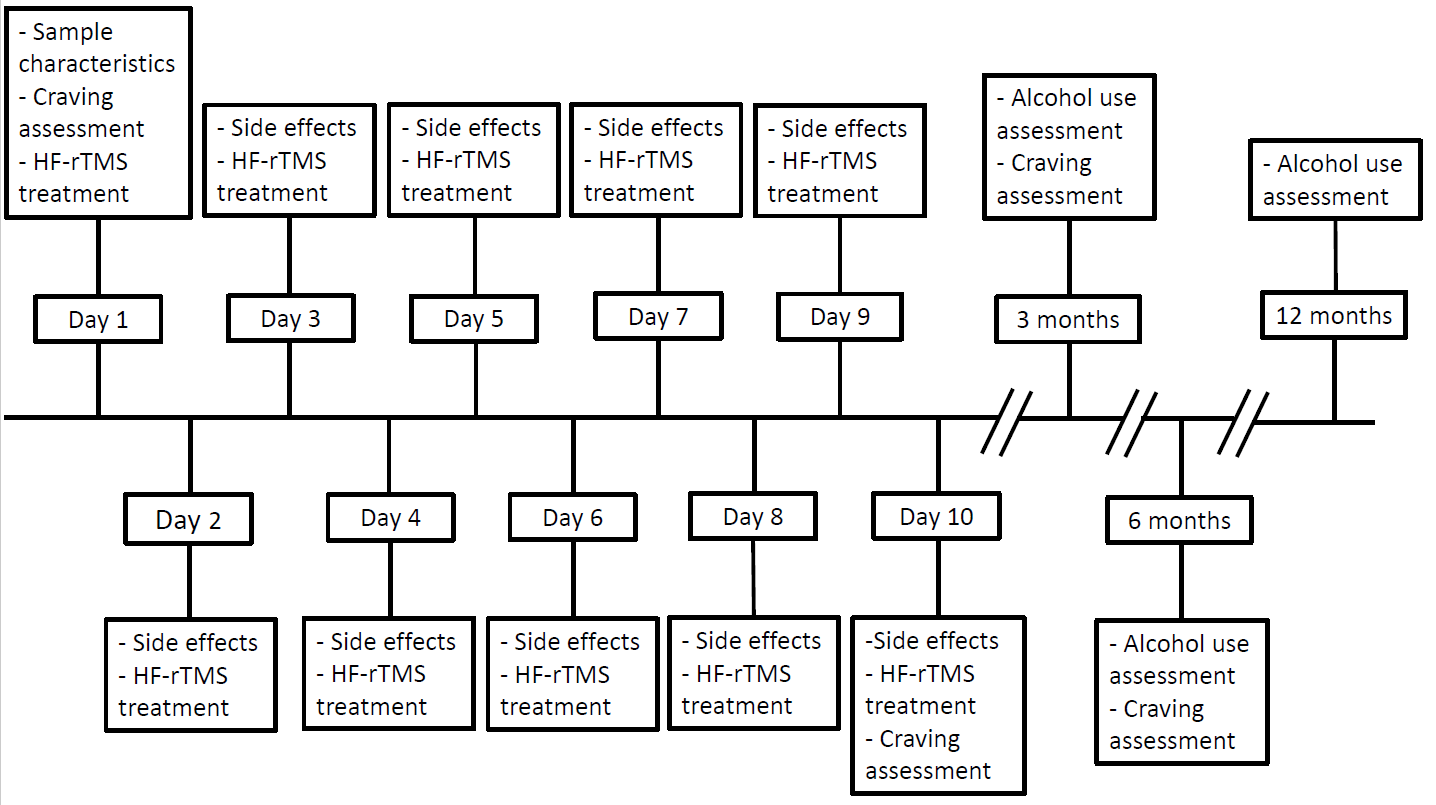


**Figure S1:** Schematic overview of the study procedure.


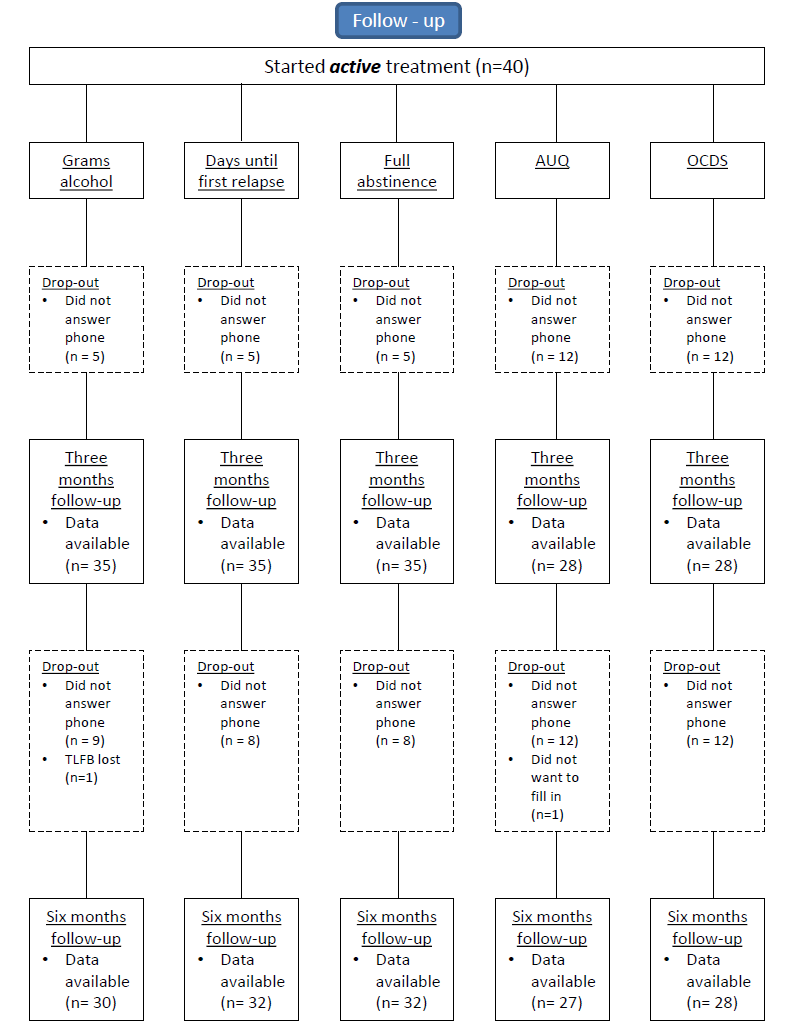


**Figure S2:** Overview of drop-out and data loss for each secondary outcome measure during follow-up for the active rTMS treatment group. Note that the numbers of available data in this flow chart do not necessarily compare to our numbers of missing data in Table 1 and 2 in the main manuscript. For example, it is possible that a subject relapsed within the first three months follow-up, which we assessed at the three months follow-up time point, and then was lost to follow-up at six months. For this outcome measure that would mean that we have complete information, but he/she would still be lost to follow-up at six months.


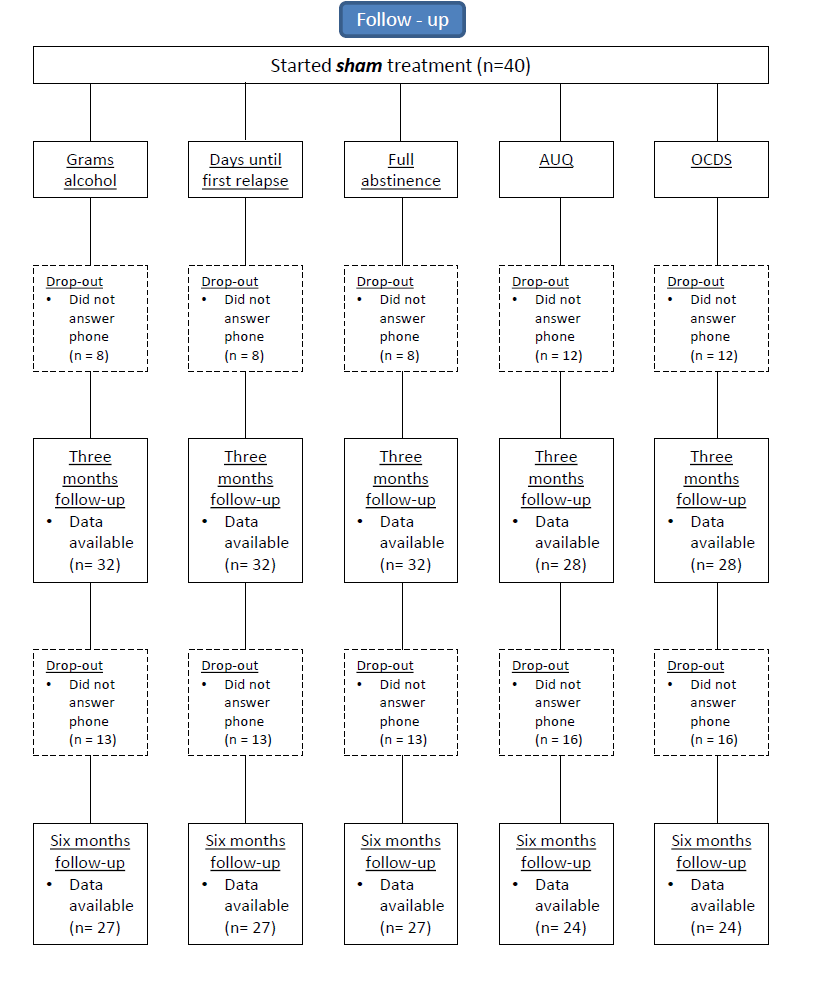


**Figure S3:** Overview of drop-out and data loss for each secondary outcome measure during follow-up for the sham rTMS treatment group.

**Table S1:** Overview of missing data patterns for the secondary alcohol use outcome measures per follow up period and treatment group. Grams alcohol, days until first relapse and full abstinence were assessed at 3 and 6 months follow-up, whereas treatment success was measured at 12 months follow up. Subjects that miss data of days until first relapse or full abstinence at 6 months did not have a relapse event and stayed abstinent for the first 3 months. NA: Not Applicable.

| **Alcohol Use Outcome Measures** | **Missing 3 month data only** | **Missing 6 month data only** | **Missing 3+6 month data** | **Missing 12 month data** |
| --- | --- | --- | --- | --- |
| **Grams alcohol** | 0 | 10  (5 Active,  5 Sham) | 13  (5 Active,  8 Sham) | NA |
| **Days until first relapse** | 0 | 2  (0 Active,  2 Sham) | 13  (5 Active,  8 Sham) | NA |
| **Full Abstinence** | 0 | 1  (0 Active,  1 Sham) | 13  (5 Active,  8 Sham) | NA |
| **Treatment succes** | NA | NA | NA | 45  (23 Active,  22 Sham) |

**Table S2:** Overview of missing data patterns for the secondary craving outcome measures per follow up period and treatment group. Craving was assessed pre-treatment, post-treatment, at 3 and 6 months follow up, resulting in various missing data patterns.

| **Craving**  **Outcome Measures** | **Missing post treatment only** | **Missing 3 month data only** | **Missing 6 month data only** | **Missing post + 3 month data** | **Missing post + 6 month data** | **Missing 3 + 6 month data** | **Missing post + 3 + 6 month data** |
| --- | --- | --- | --- | --- | --- | --- | --- |
| **AUQ** | 1  (1 Active, 0 Sham) | 9 (6 Active, 3 Sham) | 11  (5 Active, 6 Sham) | 0 | 3  (2 Active, 1 Sham) | 9  (2 Active, 7 Sham) | 6  (4 Active, 2 Sham) |
| **OCDS** | 1  (1 Active, 0 Sham) | 9 (6 Active, 3 Sham) | 10  (4 Active, 6 Sham) | 0 | 3  (2 Active,  1 Sham) | 9  (2 Active,  7 Sham) | 6  (4 Active, 2 Sham) |

**Table S3:** Side effects reported in the rTMS and sham treatment groups.

| **Side effect** | **Active group (N = 40)** | **Sham group (N= 40)** | **Test statistic** | **P-value** |
| --- | --- | --- | --- | --- |
| **Headache (%)** | 1.9 | 4.5 | Χ^2^(1) = 4.477 | 0.034 |
| **Pain or beep in the ear (%)** | 1.3 | 0 | Fisher’s exact test | 0.249 |
| **Reduced hearing (%)** | 0 | 0 | - | - |
| **Fainting (%)** | 0 | 0 | - | - |
| **Epileptic seizure (%)** | 0 | 0 | - | - |
| **Uncomfortable sensation at stimulation site (%)** | 3.5 | 0.54 | Χ^2^(1)= 4.407 | 0.036 |
| **Tiredness after stimulation (%)** | 0.27 | 0.54 | Fisher’s exact test | 1.000 |

**Additional Analysis**

We performed an additional analysis on our primary outcomes measure, in order to take into account the factor of time and check for time x group interactions. Since 9 subjects did have data on abstinent days at three months follow-up, but not at six months follow-up, we could not calculate the number of abstinent days in the 180 days after treatment. In order to take these values into account and to examine the effect of time on number of abstinent days we performed an additional generalized mixed-effects model with Poisson family in which we modeled the effect of treatment group, time (3 and 6 months) and their interaction as fixed factors. Again, we did not find a main effect of treatment group (β = 0.325 +- 0.451, Z = 0.721, p = 0.471), and no interaction effect between treatment group and time (β =-0.058 +- 0.041, Z = 1.41, p=0.157). We did find a significant main effect of time, which showed that the number of abstinent days decreased over time (β = -0.238 +- 0.030, Z = -8.052, p < 0.001).
